# Swin–UNet++: A Nested Swin Transformer Architecture for Location Identification and Morphology Segmentation of Dimples on 2.25Cr1Mo0.25V Fractured Surface

**DOI:** 10.3390/ma14247504

**Published:** 2021-12-07

**Authors:** Pan Liu, Yan Song, Mengyu Chai, Zelin Han, Yu Zhang

**Affiliations:** 1 School of Chemical Engineering and Technology, Xi’an Jiaotong University, Xi’an 710049, China; lp2441867383@stu.xjtu.edu.cn (P.L.); chaimengyu929@stu.xjtu.edu.cn (M.C.); hanzelin@stu.xjtu.edu.cn (Z.H.); 2 School of Computer Science, Shaanxi Normal University, Xi’an 710119, China

**Keywords:** artificial intelligence, dimple, ductile fracture, fractured surface, semantic segmentation, transformer

## Abstract

The precise identification of micro-features on 2.25Cr1Mo0.25V steel is of great significance for understanding the mechanism of hydrogen embrittlement (HE) and evaluating the alloy’s properties of HE resistance. Presently, the convolution neural network (CNN) of deep learning is widely applied in the micro-features identification of alloy. However, with the development of the transformer in image recognition, the transformer-based neural network performs better on the learning of global and long-range semantic information than CNN and achieves higher prediction accuracy. In this work, a new transformer-based neural network model Swin–UNet++ was proposed. Specifically, the architecture of the decoder was redesigned to more precisely detect and identify the micro-feature with complex morphology (i.e., dimples) of 2.25Cr1Mo0.25V steel fracture surface. Swin–UNet++ and other segmentation models performed state-of-the-art (SOTA) were compared on the dimple dataset constructed in this work, which consists of 830 dimple scanning electron microscopy (SEM) images on 2.25Cr1Mo0.25V steel fracture surface. The segmentation results show Swin–UNet++ not only realizes the accurate identification of dimples but displays a much higher prediction accuracy and stronger robustness than Swin–Unet and UNet. Moreover, efforts from this work will also provide an important reference value to the identification of other micro-features with complex morphologies.

## 1. Introduction

With good resistance to hydrogen damage, vanadium (V)-modified Cr–Mo steel (i.e., 2.25Cr1Mo0.25V steel) is widely applied in the fabrication of hydrogen storage vessels [1]. Nevertheless, the severe hydrogen environment with high pressure can lead to hydrogen-induced deterioration, even hydrogen embrittlement (HE) of vanadium (V)-modified Cr–Mo steel [2,3,4,5]. To improve the alloy design and resistance to HE, tracing back to the origin of structural failures is essential. Presently, there are some useful tools or techniques (i.e., optical profilometer, in-situ techniques, fracture surface topography analysis (FRASTA), and finite element analysis (FEA)) to analyze the important factors that governed the fractured process [6,7,8]. Particularly, the percentage of dimples on a fracture surface can quantitatively represent the fracture pattern (i.e., ductile fracture and brittle fracture) and further evaluate the hydrogen-induced ductility loss of 2.25Cr1Mo0.25V steel [9]. Precisely locating, segmenting, and calculating the area of the dimples with complex morphology on a 2.25Cr1Mo0.25V steel fracture surface is the precondition to calculate its percentage. However, due to the complex fracture morphology and unrecognizable boundaries of dimples, the process of manual quantification becomes cumbersome, as it requires not only substantial labor but also induces subjective bias as well [10,11,12]. In addition, during the analyses process, massive SEM images usually need to be analyzed. Therefore, it is necessary to develop a new method for precisely locating, segmenting, and calculating the areas of the dimples on the fractured surface.

Semantic segmentation is a well-known technique used for segmenting the fractured surface into different regions based on morphological characteristics. The segmentation models based on traditional machine learning techniques require experts to design the hand-crafted features for image representation [13,14,15]. These engineered features are based on the color, shape, size, or boundary information of the fractures. It is noteworthy that the performance of the hand-crafted features greatly depends on the researchers’ experience. Moreover, the process of designing these features also requires a substantial workforce.

Deep learning models [16,17,18,19,20] are an effective way of classifying and quantifying fracture characteristics [11,12,13,14,15,16,17,18,19,20,21,22,23,24]. The works presented in the literature show that the machine learning models based on convolutional neural networks (CNN) are very suitable for the detection and classification of microstructures [25,26,27,28,29,30,31]. Konovalenko et al. proposed a model to detect the edges of dimples. In addition, the authors used a CNN to estimate the size and diameter of the dimples. However, the proposed model focused on images that only contained dimples. The images of hybrid microstructures (i.e., a mixture of dimples and cleavage) were not of concern [11,32]. Recently, Sinha et al. used UNet to perform the semantic segmentation of dimples on a metallic surface. This model can well segment the clearly visible deep dimples. However, this model is inappropriate to segment the overall dimple morphology of fracture [33].

The aforementioned limitations [11,32,33] are mainly caused due to the inability of deep learning methods to model the explicit long-range relationship and the intrinsic locality of the convolution operations in CNN-based models [34]. It is notable that, usually, the distribution of the dimples is not concentrated on the fractured surface. Consequently, the model should have the ability to estimate the long-range relationship between the dimples. Moreover, the process of detecting the boundaries of dimples is a tedious task. Therefore, the ability of the model to capture the details of dimples’ boundaries is vital. However, the CNN-based encoders lose the fine details. As a result, the dimples cannot be detected precisely. The transformers are a well-known class of model used in natural language processing (NLP). These models have also been applied in various computer vision applications by the research community due to their excellent ability to model global information. The ViT model proposed by Google is based on transformers, in which the convolutional neural network was completely abandoned in the process of model construction [35]. Ze Liu et al. proposed the Swin transformer model, which realizes the hierarchical structures similar to CNN. This model is significantly computationally efficient as compared with the CNN-based models [36]. Based on the Swin transformer model, Hu cao et al. proposed the Swin–Unet model, which is the first transformer-based U-shaped architecture consisting of encoder, bottleneck, decoder, and skip connections. This model performs SOTA on publicly available medical datasets [37]. However, due to the fixed depth of the Swin–Unet model, the prediction results on different datasets have different accuracies.

In this work, based on the Swin–Unet model and UNet++ [38], we present a new model, Swin–UNet++, for precisely detecting and segmenting the dimples on the fractured surface. In the proposed Swin–UNet++ model, the decoder of the Swin–Unet is redesigned to fuse additional feature representations. A vice path is introduced to up-sample the feature maps of the last Swin transformer block of the encoder and the main path to up-sample the feature maps obtained from the bottleneck. Please note that the vice path not only provides additional semantic representations for the main path to fuse features at different levels but also preserves more detailed information of the input images. Consequently, the proposed model achieves a higher semantic segmentation accuracy compared with Swin–Unet.

The Swin–UNet++ proposed in this work successfully overcomes the limitations of the aforementioned methods. The proposed model not only detects the dimples accurately in the fractured surface but also identifies the entire dimples with complex morphology precisely. Moreover, the proposed model can be easily extended to improve the identification results for additional complex fracture morphologies. Additionally, the proposed methods can be integrated into the engineering failure analysis methods as a user-independent, accurate, and computationally efficient tool.

## 2. Methods

### 2.1. Network Architecture

**Figure 1:**
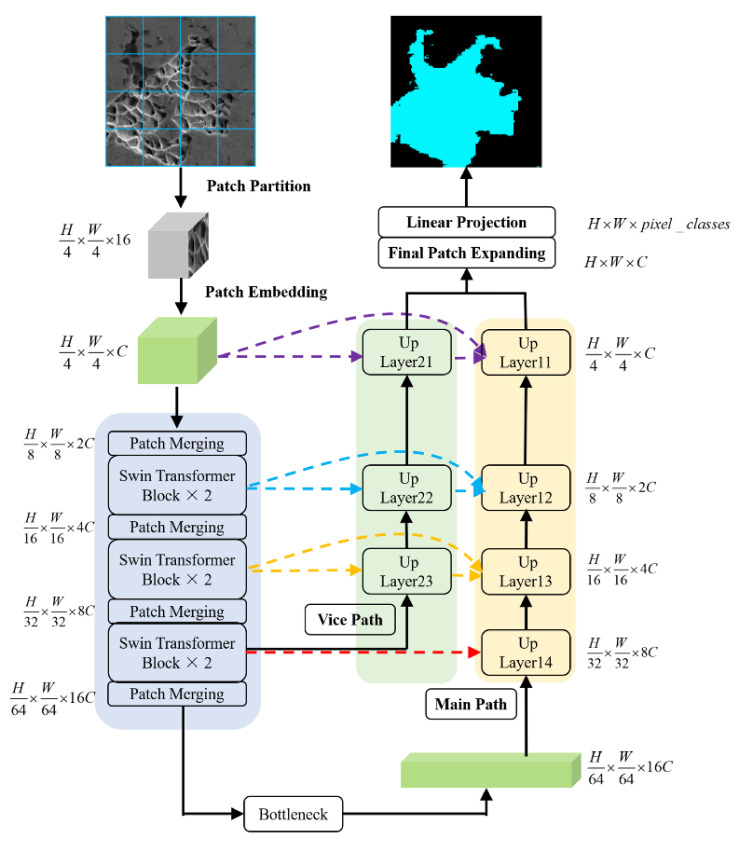
The architecture of Swin–UNet++.

The architecture of the proposed network is presented in Figure 1. The proposed network comprises an encoder, bottleneck, and decoder. In the encoder, we divide the input image into 4 × 4 non-overlapping patches by using the patch partition layer. Then, these patches are converted to sequence embeddings. Given that the dimple images acquired using the SEM are grayscale (single-channel) images, the feature dimension of each patch is 4 × 4 × 1 = 16. These feature dimensions are projected on the appropriate dimension, which is a hyper-parameter C in the embedding layer. These patches pass through the structure four times, where the Swin transformer blocks are used to learn the feature representations. The patch merging layer is used to down-sample the patches and generate the hierarchical feature representations.

Inspired by the UNet++ architecture and based on the patching expanding layer proposed in Swin–Unet in the decoder architecture, we introduce a vice path to up-sample the feature representations from the last Swin transformer block of the encoder. This enables us to preserve more detailed information of the input images and provide additional shallow features for the main path to fuse. Finally, the up-sampled features generated by the decoder are linearly projected to produce the prediction results on a pixel level. The details of the decoder architecture are discussed in the following section.

### 2.2. Swin Transformer Block

**Figure 2:**
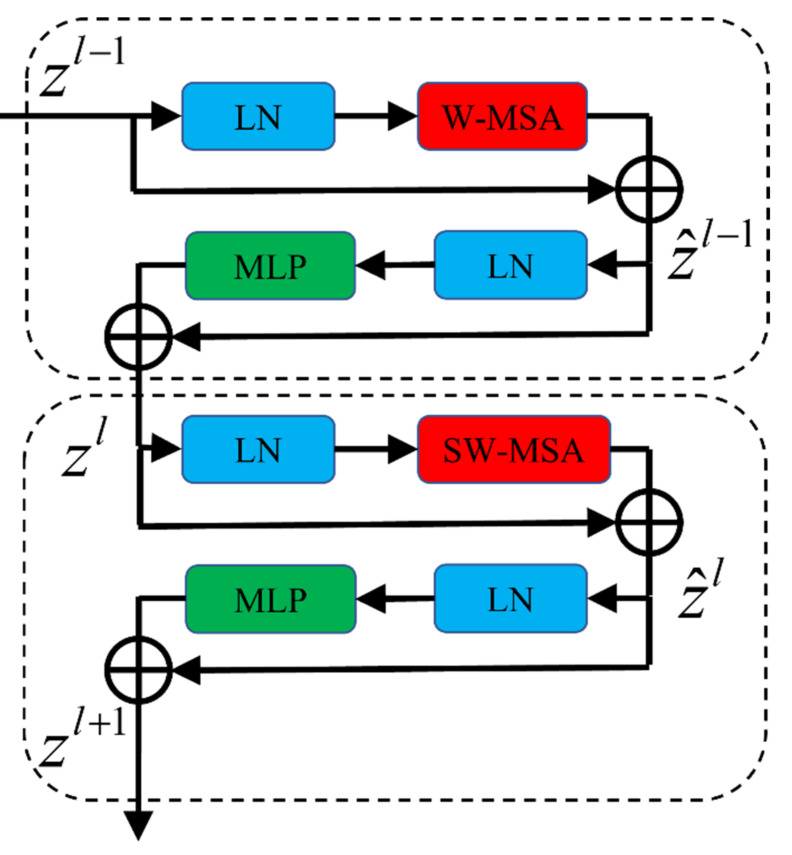
Swin transformer block.

As compared with the traditional transformer’s multi-attention mechanism, the Swin transformer block uses a shafted window to reduce the computational cost during the process of modeling relationships between patches. This modification allows us to use the transformer architecture in a wide range of computer vision applications. The architecture of the Swin transformer block is illustrated in Figure 2. It consists of two consecutive transformer blocks. The first block is applied to a window-based multi-head self-attention (W-MSA), and the latter is applied to a shafted window-based multi-head self-attention (SW-MSA) to compute the relationships between patches.

### 2.3. Decoder

**Figure 3:**
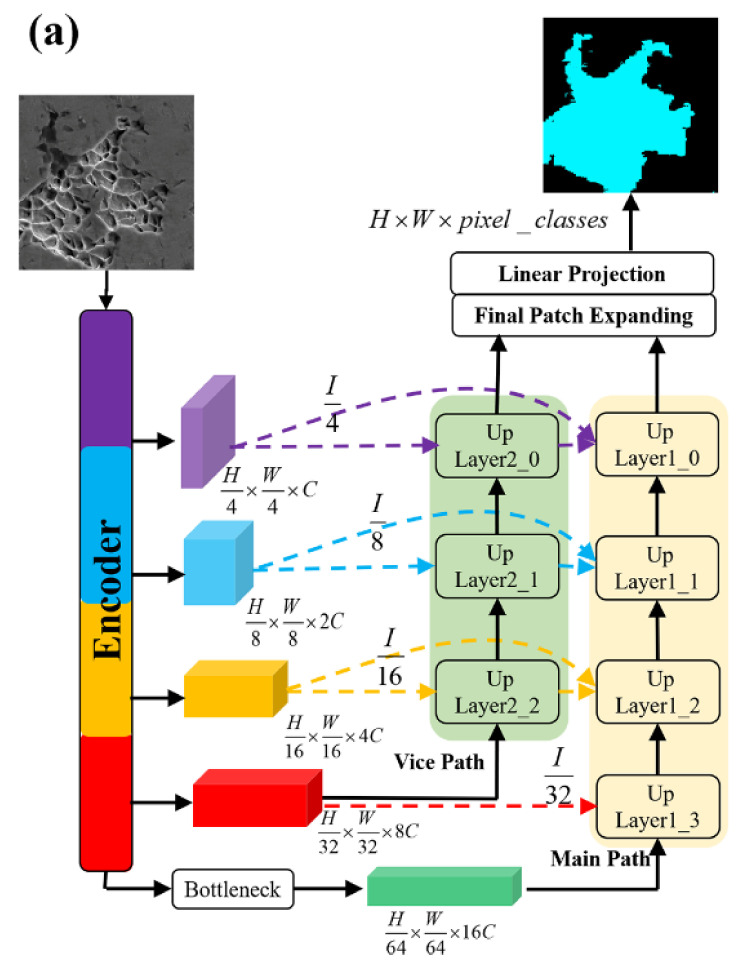

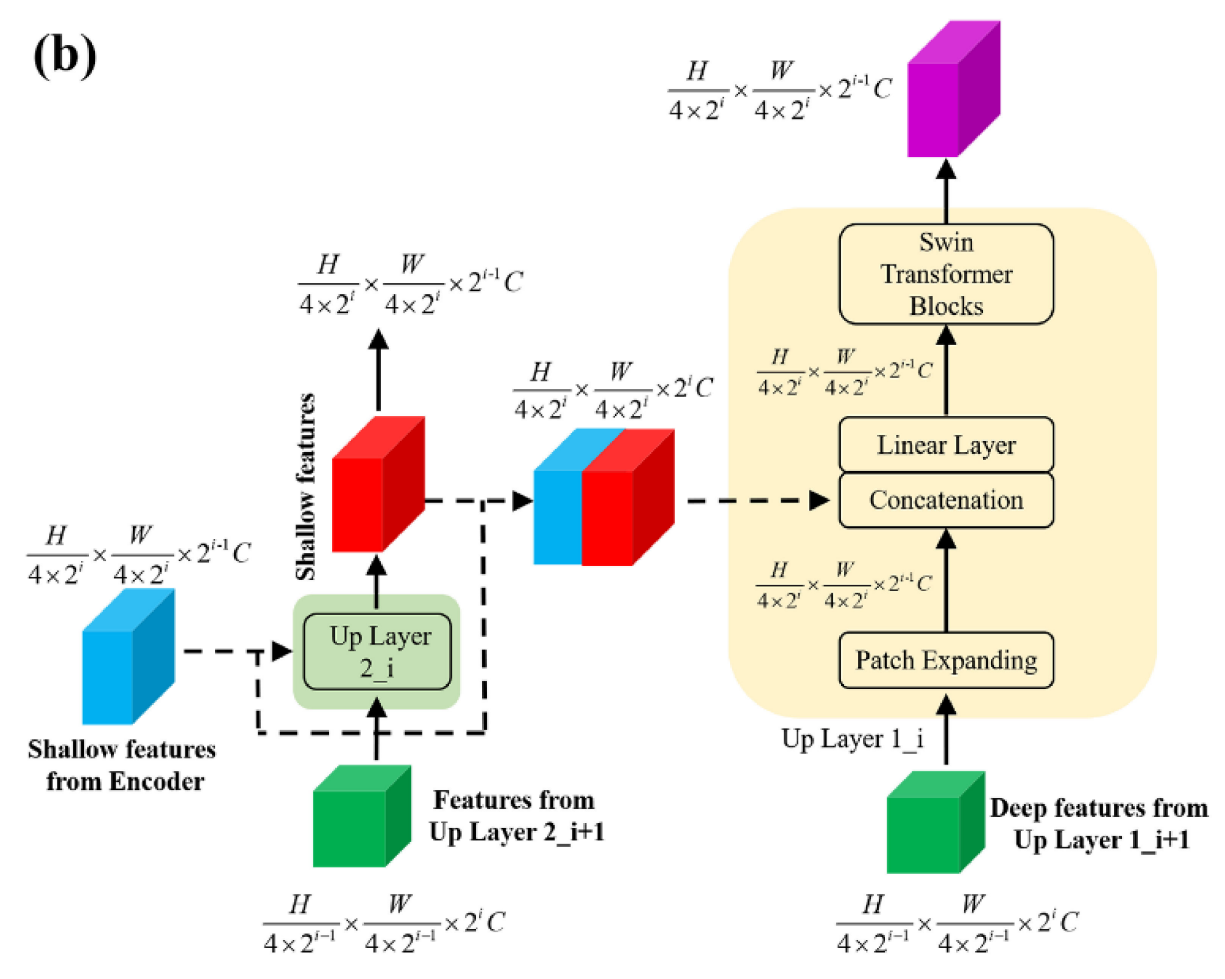
(**a**) The architecture of decoder in Swin–UNet++. (**b**) The presentation of feature fusion and up-sampling process in decoder.

In this work, we redesigned the architecture of the decoder in Swin–Unet to fuse additional feature representations. As compared with Hu cao’s Swin–Unet model [37], seen in Figure 3a, the proposed decoder consists of a vice path and main path to up-sample the feature representations learned by the encoder and bottleneck. The vice path is introduced to up-sample the feature maps obtained from the last Swin transformer block of the encoder, and the main path is introduced to up-sample the information obtained from the bottleneck. In the skip connection, the vice path enables us to consider additional semantic representations to be fused by the main path.

In Swin–Unet, the skip connection fuses one shallow feature and one deep feature. In contrast, in this work, there are two shallow features and one deep feature fused in the up-layer module of the main path. In addition, the vice path provides additional details regarding the input image. As a result, the loss of spatial information caused due to down-sampling is reduced. As presented in Figure 3b, the hierarchical feature representations learned by the encoder and the bottleneck are fed to the decoder to restore the spatial resolution. Each up-layer module transforms the dimensions of the input feature from ($\frac{H}{4\times 2^{i-1}}\times \frac{W}{4\times 2^{i-1}}\times 2^{i}C$) to ($\frac{H}{4\times 2^{i}}\times \frac{W}{4\times 2^{i}}\times 2^{i-1}C$) by using a patch-expanding module and liner layer. The depth of the up-layer module is denoted as i. Finally, through a series of feature fusions, up-sampling, and linear mapping processes, the semantic representations at different levels are transformed to pixel-level prediction results.

## 3. Results and Discussion

### 3.1. Datasets

**Figure 4:**
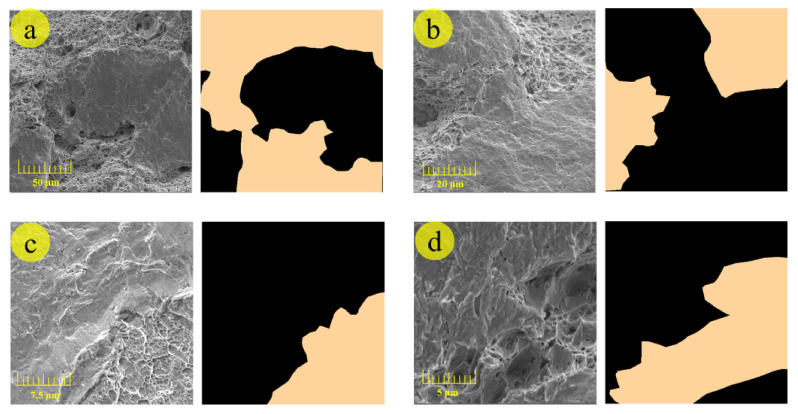
Dimples at different scales (from (**a**) to (**d**), the resolution is elevated gradually) and their annotated label used in model training. The black area in the annotated label represents the background, and the red area is considered dimples.

To acquire SEM images of the fracture surface and various dimples’ morphology as rich as possible, the fracture surfaces of our previous tensile experiments of 2.25Cr1Mo0.25V steel, performed under different conditions (i.e., different strain rates, heat treatment conditions, and positions of specimens), were observed with the aid of SEM (MAIA3LMH, TESCAN, Czech Republic) for constructing the training dataset. In addition, all images were taken at a resolution of 1280 × 960 pixels, which were cropped to a 512 × 512-pixel size on account of the limitation of GPU memory. The final dataset was composed of 604 training images and 226 test images that contained various dimples with complex morphology at different scales. As shown in Figure 4, the shallow dimples are dominant in (a) and (c), while deep dimples are superior in (b) and (d).

### 3.2. Training Details

We use Pytorch 1.8.1 in Ubuntu to train and test the proposed model on NVIDIA TITAN XP 12GB with CUDA 11.2. During the training process, we performed data augmentation, such as image flipping and rotations, to increase the diversity of the dataset. The size of the input image and the batch size are 512 × 512 and 4, respectively. We used an SGD optimizer with a momentum of 0.9 and weight decay of $1.0\times 10^{-4}$ to optimize the proposed model. Additionally, we set the initial value of learning rate to 0.001, which decays exponentially.

### 3.3. Training and Segmentation Results

In order to evaluate the performance of the proposed model and other models presented in the literature, the DSC (dice similarity coefficient) and HD95 (95% Hausdorff distance) were used as the evaluation metrics [37]. Specifically, the DSC metric can be calculated by the formula (1):

(1) $$DSC\;=\;\frac{2TP}{FP\;+\;2TP\;+\;FN}$$

where *TP*, *FP*, and *FN* are the numbers of true positive, false positive, and false negative pixels. HD95 metric is the 95th-percentile Hausdorff distance [39].

$$Hausdorff\;distance\;=\;max\left\{\underset{g\in G}{\max}\;\underset{p\in P}{\min}\;d\left(g,p\right),\underset{p\in P}{\max}\;\underset{g\in G}{\min}\;d\left(p,g\right)\right\}$$

where *p* denotes the set of prediction results generated by the network, *g* denotes the set of ground truth, and $d\left(p,g\right)$ indicates the Euclidean distance between pixels *p* and g [40].

The DSC represents the overlap between the region predicted by the network and the ground truth. HD95 represents the maximum distance between the region predicted by the network and the ground truth. Thus, a higher DSC and lower HD95 indicate a better performance of the semantic segmentation model. Please note that DSC is sensitive to the interior part of the segmented region and the real region, while HD95 focuses on the boundary of the segmentation result. Therefore, the combination of these two indicators allows us to objectively evaluate the segmentation performance of the models quantitatively.

Table 1 presents the comparison results of the proposed Swin–UNet++ with various state-of-the-art models presented in the literature using the dimples dataset of 2.25Cr1Mo0.25V steel constructed in this work. It is worth mentioning that the Swin–Unet and UNet were tested using the official source code.

**Figure 5:**
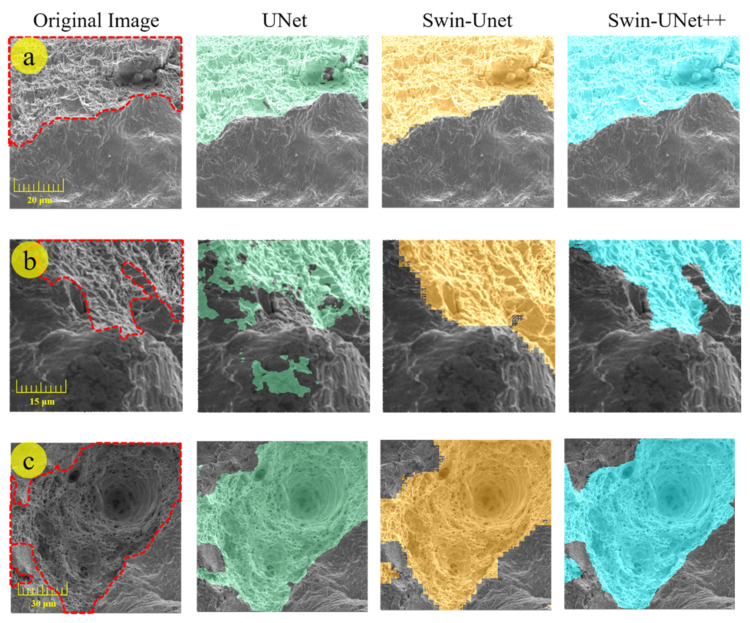

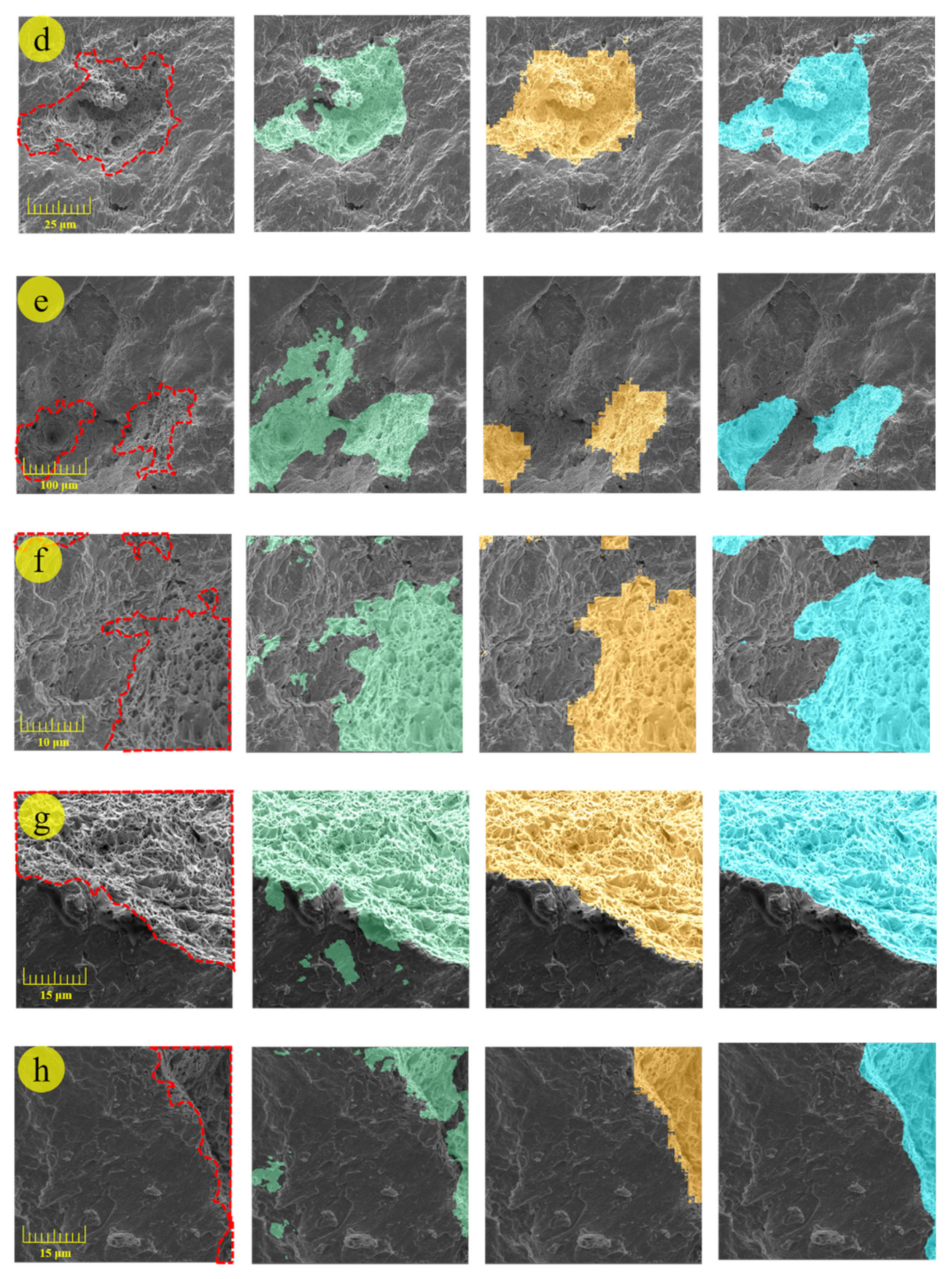
Segmentation results of different models for 8 images (**a**–**h**) selected from the test dataset constructed in this work. The boundary of dimples in original images is annotated by red dotted lines.

Results in Table 1 demonstrate that Swin–UNet++ proposed in this work achieves the best performance with a segmentation accuracy of 94.65% (DSC ↑) and 22.99 (HD95 ↓). Moreover, the results also show that the segmentation model based on the Swin transformer block performs much better than the model based on the convolution block. The DSC evaluation metric improves from 85.37% to 94.65%, and HD95 decreases from 85.76 to 22.99. Sinha’s work realized the segmentation of the obvious deep regions in dimples [33]. Different from Sinha’s model, seen in Figure 5c,f, Swin–UNet++ could detect dimples accurately and also segment the entire morphology of dimples precisely on the fracture surface.

The segmentation results also show that the proposed Swin–UNet++ performs efficiently on both the boundaries and interior regions of the dimples as compared with the CNN-based models. As compared with the UNet, the proposed Swin–UNet++ obtains significantly precise boundaries of dimples and the predicted region has no overlapping problem as well. This is mainly because the proposed Swin–UNet++ is based on the Swin transformer, which easily learns the global and long-range semantic information and preserves additional details of the input images.

Furthermore, as compared with Swin–Unet, the proposed Swin–UNet++ also shows a significant improvement in terms of evaluation metrics, i.e., the DSC increases 7.7 percentage points, and HD95 decreases from 59.33 to 22.99, which means Swin–UNet++ performs much better on the boundaries of dimples prediction and simultaneously on the interior region of dimples prediction. As presented in Figure 5e–g, the dimples boundaries generated by Swin–UNet++ are clearer and more consistent with the ground truth compared with Swin–Unet. Additionally, as seen in Figure 5b–h, Swin–UNet++ also performs better in terms of identifying the region of the dimple, which means higher accuracy in terms of area statistics. This is mainly because of the introduction of the vice path, which preserves more detailed information of the input images and provides additional shallow features to be fused by the main path.

### 3.4. Dimples Area Calculation

According to the percentage of dimples on the fracture surface, the fracture pattern (i.e., ductile fracture and brittle fracture) of 2.25Cr1Mo0.25V steel can be quantitatively analyzed. In addition, the hydrogen-induced ductility loss and deterioration can also be evaluated quantitatively. Consequently, it is vital to develop a method for accurately calculating the dimples’ area. However, due to the complexity of dimple morphology, there are few studies on accurate dimple area calculation based on image recognition. In this work, considering the dimples’ segmentation results obtained by using the proposed Swin–UNet++, it is very convenient to calculate the dimples’ area. It is notable that the proposed method avoids subjective deviations in the artificial statistical dimples and is computationally efficient as well.

**Figure 6:**
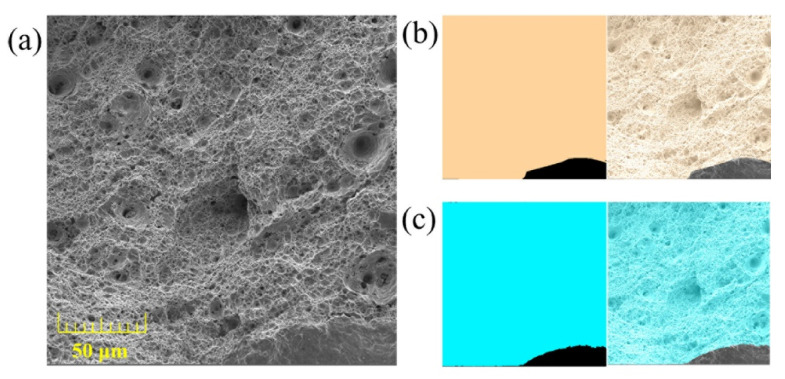
(**a**) The original fracture image; (**b**) dimple region with burlywood color is annotated manually; (**c**) dimple region with blue color is predicted by Swin–UNet++.

As presented in Figure 6b, the region with the burlywood color represents the manually annotated dimple, whose area denotes the ground truth (true area). The blue region presented in Figure 6c represents the dimple predicted by the proposed Swin–UNet++. For the image presented in Figure 6a, the true area is 0.9502, and the predicted area is 0.9495. The corresponding relative error is 0.07367%.

**Figure 7:**
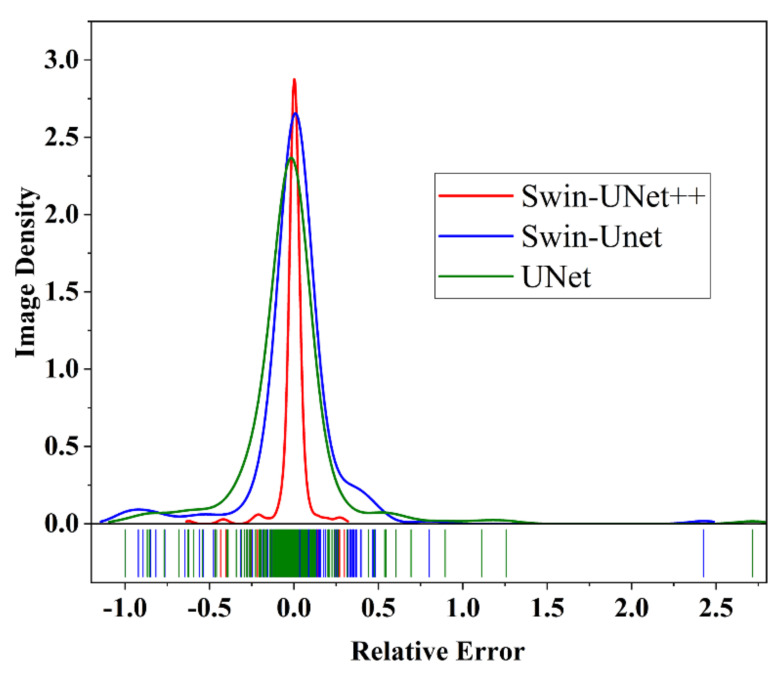
The distribution of relative error between true area and prediction area generated by different models.

Based on the 226 dimple SEM images available in the test set, we analyzed the relative error between the true area and the predicted area of the dimples. The resulting distribution and statistic metrics are presented in Figure 7 and Table 2. As compared with Swin–Unet and UNet, the relative error of the proposed Swin–UNet++ has a significantly lower mean (0.03386, 73.85% ↓), standard deviation (0.0738, 70.89% ↓), and maximum value (0.6230, 74.32% ↓). This illustrates that the stability and robustness of the areas of the predicted dimples based on the proposed Swin–UNet++ are better. This enhances the reliability of the computed area of the dimples having complex morphology in an automatic way.

**Figure 8:**
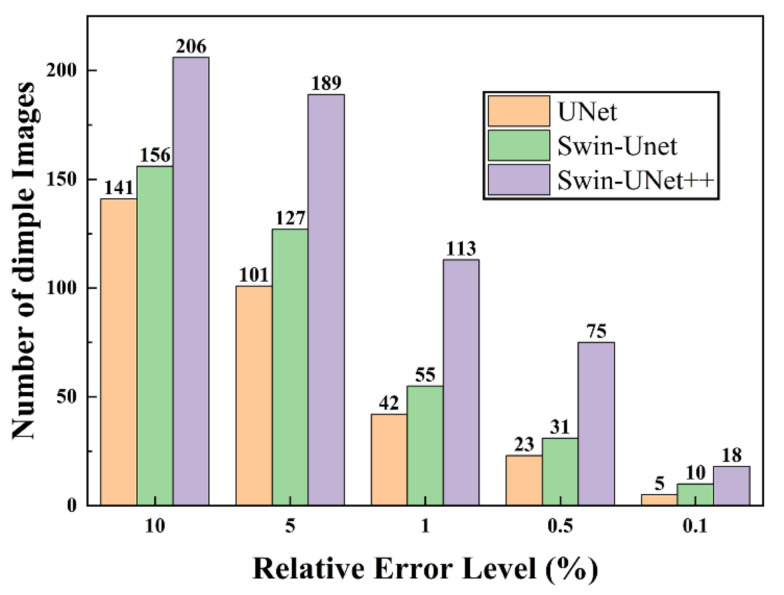
Number of dimple SEM images that reached different relative error levels.

In order to clearly present the advantages of the proposed Swin–UNet++, various images with different relative errors generated using the proposed and other methods are presented in Figure 8. As compared with the UNet and Swin–Unet, the results presented in Figure 8 illustrate that the proposed Swin–UNet++ performs significantly better in terms of the predicted area of the dimples on the fracture surface.

For the 226 dimple images in the test set, there are 206, 156, and 141 images obtained by using the proposed method, Swin–Unet, and UNet, respectively, which have a relative error of less than 10%. Similarly, there are 189, 127, and 101 images obtained using the proposed method, Swin–Unet, and UNet, respectively, for which the relative error is less than 5%.

## 4. Conclusions

In this work, a new segmentation model, Swin–UNet++, was proposed to perform the automatic representation of micro-features (i.e., dimples) of an alloy fracture surface with complex morphology. Based on Swin–Unet, we redesigned the architecture of the decoder to fuse additional feature representations. We introduced a vice path to up-sample the feature maps obtained from the last Swin transformer block of the encoder and a main path to up-sample the information obtained from the bottleneck. This efficiently reduces the loss of spatial information caused by down-sampling. Based on the dimple dataset collected in this work, various semantic segmentation models, including the proposed Swin–UNet++, Swin–Unet, and UNet were trained. The segmentation results show that as compared with the CNN-based models, the proposed Swin–UNet++ shows a great improvement in terms of DSC (from 85.47% to 94.65%) and HD95 (from 85.76 to 22.99) evaluate metrics. Similarly, as compared with Swin–Unet, the performance of the proposed Swin–UNet++ shows evident improvement in terms of DSC (from 86.95% to 94.65%) and HD95 (from 59.33 to 22.99). The proposed model not only segments the dimples with distinguishable features, such as deep dimples, but also segments the indiscernible dimples, such as tiny dimples and dimples with unclear boundaries. In addition, based on the proposed Swin–UNet++, the areas of dimples with complex morphology were also analyzed. The results show that there are 189 dimple images whose relative error is less than 5%.

The proposed model, Swin–UNet++, uses a transformer to analyze the dimples of an alloy’s fractured surface and achieves a high prediction accuracy in terms of location identification, morphology segmentation, and area calculation. The accurate prediction results can provide a useful and objective criterion for dimples recognition with complex morphology and unclear boundaries. It is of great significance for the quantitative analysis of hydrogen-induced ductility loss and deterioration. Generally, the idea of designing the decoder architecture in this work can also be applied to improve the performance of other neural network models. Moreover, it is noteworthy that Swin–UNet++ can be extended conveniently to improve the location identification and morphology segmentation accuracy of micro-features with complex morphologies as well and easily integrated into the engineering failure analysis processes as a user-independent, accurate, and computationally efficient tool.

## Figures and Tables

**Table 1:** Segmentation accuracy of different models on dimples dataset constructed in this work.

Model	DSC/%	HD95
UNet	85.47	85.76
Swin–Unet	86.95	59.33
Swin–UNet++	94.65	22.99

**Table 2:** Statistics of relative error between true area and prediction area generated by different models.

Models	Number of Images	Mean	Standard Deviation	Maximum
UNet	226	0.1472	0.2736	2.716
Swin–Unet	226	0.1295	0.2524	2.426
Swin–UNet++	226	0.03386	0.07348	0.6230

## Data Availability

The data used to support the findings of this study are available from the corresponding author upon request.

## Nomenclature

CNN	Convolution neural network
HE	Hydrogen embrittlement
SOTA	State-of-the-art
NLP	Natural language processing
MLP	Multi-layer perception
LN	Layer normalization
DSC	Dice similarity coefficient
HD95	95% Hausdorff distance
H	Height of input picture
W	Width of input picture
C	The output dimensions of embedding layer
